# Vaccinia Virus Inhibits NF-κB-Dependent Gene Expression Downstream of p65 Translocation

**DOI:** 10.1128/JVI.02627-13

**Published:** 2014-03

**Authors:** Rebecca P. Sumner, Carlos Maluquer de Motes, David L. Veyer, Geoffrey L. Smith

**Affiliations:** Department of Pathology, University of Cambridge, Cambridge, United Kingdom

## Abstract

The transcription factor nuclear factor kappa light-chain enhancer of activated B cells (NF-κB) plays a critical role in host defense against viral infection by inducing the production of proinflammatory mediators and type I interferon. Consequently, viruses have evolved many mechanisms to block its activation. The poxvirus vaccinia virus (VACV) encodes numerous inhibitors of NF-κB activation that target multiple points in the signaling pathway. A derivative of VACV strain Copenhagen, called vv811, lacking 55 open reading frames in the left and right terminal regions of the genome was reported to still inhibit NF-κB activation downstream of tumor necrosis factor alpha (TNF-α) and interleukin-1β (IL-1β), suggesting the presence of one or more additional inhibitors. In this study, we constructed a recombinant vv811 lacking the recently described NF-κB inhibitor A49 (vv811ΔA49), yielding a virus that lacked all currently described inhibitors downstream of TNF-α and IL-1β. Unlike vv811, vv811ΔA49 no longer inhibited degradation of the phosphorylated inhibitor of κBα and p65 translocated into the nucleus. However, despite this translocation, vv811ΔA49 still inhibited TNF-α- and IL-1β-induced NF-κB-dependent reporter gene expression and the transcription and production of cytokines induced by these agonists. This inhibition did not require late viral gene expression. These findings indicate the presence of another inhibitor of NF-κB that is expressed early during infection and acts by a novel mechanism downstream of p65 translocation into the nucleus.

## INTRODUCTION

The transcription factor nuclear factor kappa light-chain enhancer of activated B cells (NF-κB) is often activated upon viral infection of cells and plays a key role in antiviral immunity by regulating the expression of a myriad of proinflammatory cytokines and chemokines, as well type I interferon (IFN) (1). To evade innate immunity, viruses must therefore prevent the activation of NF-κB, and this is achieved in multiple ways (2). Vaccinia virus (VACV), a member of the poxvirus family of large DNA viruses and the vaccine used to eradicate smallpox (3), expresses many proteins that inhibit the activation of the innate immune response and devotes many proteins to the dampening of NF-κB activation (4, 5). Discovering novel viral inhibitors of NF-κB not only provides a greater understanding of the immune response to infection but also may aid in the design of novel anti-inflammatory therapeutics (6).

NF-κB is activated downstream of multiple pattern recognition receptors (PRRs), involving different signaling proteins depending on the PRR. Engagement of tumor necrosis factor alpha (TNF-α) with its cognate receptor on the cell surface induces an intracellular signaling cascade comprising the adaptor proteins tumor necrosis factor receptor-associated factor 2 (TRAF2) or TRAF5, whereas signaling downstream of interleukin-1β (IL-1β) and the Toll-like receptors (TLRs) utilizes TRAF6. Activation of the two signaling pathways induces TRAF-mediated formation of lysine-63- and methionine-1-linked ubiquitin chains, which are recognized by the transforming growth factor beta-activated kinase 1 (TAK1) complex and the inhibitor of κB (IκB) kinase (IKK) complex, respectively (7). Simultaneous recruitment of these complexes facilitates TAK1-dependent activation of the IKK catalytic subunits (IKKα and IKKβ), which phosphorylate IκB (8, 9). In resting cells, IκBα is found in complex with NF-κB transcription factor subunits p65 and p50, preventing their nuclear translocation and activation of NF-κB-dependent gene transcription. Following phosphorylation, IκBα becomes ubiquitinated by an E3 ligase complex consisting of β-transducing repeat-containing protein (β-TrCP) (10) and is subsequently degraded by the proteasome, thus releasing p65/p50 into the nucleus and allowing transcription to occur.

To date, VACV has been described to encode nine intracellular inhibitors of NF-κB activation downstream of the TNF-α and IL-1β receptor and TLRs. Proteins A46, A52, and K7 exert their inhibitory activity close to the receptor complexes by interacting with upstream signaling adaptor molecules. A46 interacts with several Toll–IL-1 receptor (TIR) domain-containing proteins, including myeloid differentiation primary response gene 88 (MyD88), TIR adaptor protein (TIRAP), TIR-domain-containing adaptor-inducing beta interferon (TRIF), and TRIF-related adaptor molecule (TRAM), allowing it to inhibit NF-κB activation downstream of multiple PRRs (11, 12). Due to its interaction with TRIF, it is also an inhibitor of IFN regulatory factor 3 (IRF-3) (11). Both A52 and K7 interact with IL-1 receptor-associated kinase 2 (IRAK2) and TRAF6, thus inhibiting downstream of TLRs and IL-1β but not TNF-α (13–15). Acting further downstream in the signaling cascade, B14 binds to IKKβ and inhibits phosphorylation on its activation loop (16), and N1 has also been described to target the IKK complex (17), although this interaction was later disputed (16). Despite uncertainty about the mechanism, the NF-κB-inhibitory activity of N1 has been confirmed in several studies (18–20). C4 was described to inhibit NF-κB activation at the level of or downstream of the IKK complex, although the exact mechanism is unknown (21). Lastly, protein K1 has been described to block IκB degradation (22), and protein M2 reduces the extracellular signal-regulated kinase 2 (ERK2) phosphorylation induced by phorbol myristate acetate, preventing the translocation of p65 into the nucleus (23). Recently, protein A49 was added to this list and shown to be a novel virulence factor that targets β-TrCP (24). In the presence of A49, even if IκBα is phosphorylated, it is not ubiquitinated or degraded by the proteasome, thus remaining bound to NF-κB in the cell cytoplasm.

In addition to the inhibitors detailed above, it was reported earlier that several other orthopoxviruses express NF-κB inhibitors (25), and in some cases, these are not present in VACV. These include cowpox virus (CPXV) protein CP77, which attenuates NF-κB activation by binding to the NF-κB subunit p65 and also to Skp1 and cullin 1 of the E3 ubiquitin ligase complex (26). Furthermore, variola virus protein G1, which is conserved in several orthopoxviruses, including CPXV, monkeypox virus, and ectromelia virus, but not VACV, interacts with the NF-κB subunit p105 and Skp1 (27).

Viruses with large regions of the genome deleted are useful tools in the discovery of novel molecules involved in the modulation of cellular responses. This is particularly helpful in the case of large DNA viruses, in which functional redundancy might mask the effect of a given protein. The deletion mutant vv811, based on the Copenhagen strain of VACV, has 55 open reading frames (ORFs) deleted from the left and right terminal regions of the genome (28), areas that are rich in immunomodulatory proteins (29). This virus, however, was found to accumulate the phosphorylated form of IκBα (pIκBα) and to inhibit NF-κB activation downstream of both TNF-α and IL-1β, despite, at that time, lacking all known NF-κB inhibitors of these pathways (30). This enabled the authors to conclude that a further inhibitor(s) exists in the genome (30).

In this study, we constructed a derivative of vv811 lacking the recently described NF-κB inhibitor A49 (vv811ΔA49) (24). Unlike vv811-infected cells, cells infected with vv811ΔA49 no longer accumulate pIκBα, consistent with the role of A49 in interacting with the E3 ligase β-TrCP, inhibiting the ubiquitination and subsequent degradation of IκBα. Following the degradation of IκBα in response to either TNF-α or IL-1β stimulation of vv811ΔA49-infected cells, p65 translocated into the nucleus, indicating that no further proteins that prevent p65 translocation are expressed by this virus. However, despite p65 translocation, infection with vv811ΔA49 still prevented the TNF-α- and IL-1β-induced activity of an NF-κB reporter and inhibited the transcription and production of cytokines induced by these agonists. This inhibitory activity did not require late viral gene expression. These data indicate that another protein or proteins expressed early during infection are able to inhibit NF-κB reporter activity and dampen the expression of a number of TNF-α- and IL-1β-induced proinflammatory genes.

## MATERIALS AND METHODS

### Cells, viruses, and reagents.

HEK293T, BSC-1, A549, and CV-1 cells were grown in Dulbecco's modified Eagle's medium (DMEM; Gibco) supplemented with 10% heat-treated (56°C, 1 h) fetal bovine serum (FBS; Biosera) and penicillin-streptomycin (50 μg/ml). A549-NF-κB-LUC cells were constructed by transducing A549 cells with a lentivirus encoding firefly luciferase (LUC) under the control of an NF-κB promoter (obtained from David Escors, University College London, London, United Kingdom). Parental vv811 was obtained from Michelle Barry (University of Alberta, Canada). The strain of Western Reserve (WR) lacking *A49R* has been described previously (24). TNF-α and IL-1β were both obtained from Peprotech.

### Construction of vv811 lacking *A49R*.

For construction of the vv811 A49 deletion virus (vv811ΔA49), a plasmid containing the 300-bp flanking regions of the *A49R* gene and the Escherichia coli guanylphosphoribosyl transferase (*Ecogpt*) gene fused in-frame with the *EGFP* gene (pΔA49) (24) was used. vv811ΔA49 was constructed using the transient dominant selection method (31) by selection of enhanced green fluorescent protein (EGFP)-positive plaques in the presence of mycophenolic acid, hypoxanthine, and xanthine, as described previously (32). The genotype of the resolved viruses was analyzed by PCR following proteinase K treatment of infected BSC-1 cells using primers that had been used to construct pΔA49 and anneal to the flanking regions of *A49R* (24).

### SDS-PAGE and immunoblotting.

For immunoblot analysis, cells were lysed in a cell lysis buffer containing 50 mM Tris, pH 8, 150 mM NaCl, 1 mM EDTA, 10% (vol/vol) glycerol, 1% (vol/vol) Triton X-100, and 0.05% (vol/vol) NP-40 supplemented with protease inhibitors (Roche). Proteins were then quantified using a bicinchoninic acid protein assay kit (Pierce). For immunoblotting of phosphorylated proteins, the cell lysis buffer was also supplemented with phosSTOP phosphatase inhibitor cocktail tablets (Roche), and samples were subjected to SDS-PAGE immediately after lysis. The following primary antibodies were from the indicated sources: mouse anti-α-tubulin, Upstate Biotech; mouse anti-pIκBα (S32/36), Cell Signaling Technology; mouse anti-total IκBα (tIκBα), a kind gift from Ron Hay, University of Dundee, Dundee, United Kingdom; mouse anti-p65, Santa Cruz; and mouse anti-lamin A/C, Abcam. Mouse anti-D8 monoclonal antibody AB1.1 (33) and the rabbit anti-A49 polyclonal antiserum (24) have been described previously. Primary antibodies were detected with goat anti-mouse/rabbit IRdye 800CW infrared dye secondary antibodies, and membranes were imaged using an Odyssey infrared imager (LI-COR Biosciences).

### Single-step growth analysis.

To analyze viral replication over 24 h, A549 cells were infected on ice for 90 min in duplicate with the vv811 recombinant viruses or Copenhagen at 5 PFU per cell. The cells were then washed twice with medium to remove unbound virus, fresh medium was added, and the cells were transferred to a 37°C incubator. At 0 h and 24 h postinfection, the cells were scraped in their medium and were collected by centrifugation at 500 × *g* for 5 min. Cells were subjected to three rounds of freeze-thawing before the infectious viral titer was determined by plaque assay on BSC-1 cells.

### Plaque size analysis.

BSC-1 cells were infected with vv811 recombinant viruses or Copenhagen at 50 PFU per well for 5 days. The cells were washed once with phosphate-buffered saline (PBS) and stained for 1 h with crystal violet (5% [vol/vol] crystal violet solution [Sigma], 25% [vol/vol] ethanol). The sizes of 10 plaques per well were measured using Axiovision acquisition software and a Zeiss AxioVert.A1 inverted microscope, as described previously (34).

### Immunofluorescence.

For immunofluorescence microscopy, A549 cells were seeded into six-well plates containing sterile glass coverslips. Following infection with vv811 recombinant viruses and stimulation with TNF-α or IL-1β, the cells were washed twice with ice-cold PBS and fixed in 4% (vol/vol) paraformaldehyde. The cells were then quenched in 150 mM ammonium chloride, permeabilized in 0.1% (vol/vol) Triton X-100 in PBS, and blocked for 30 min in 5% (vol/vol) FBS in PBS. The cells were stained with mouse anti-p65 antibody (Santa Cruz) for 1 h, followed by incubation with donkey anti-mouse Alexa Fluor 488 secondary antibody (Invitrogen Molecular Probes). Coverslips were mounted in Mowiol 4-88 (Calbiochem) containing DAPI (4′,6-diamidino-2-phenylindole). Images were taken on a Zeiss LSM780 confocal microscope using Zen2011 acquisition software.

### Cell fractionation.

A549 cells that had been infected with the vv811 recombinant viruses and stimulated with TNF-α or IL-1β were separated into nuclear and cytoplasmic components using an NE-PER nuclear and cytoplasmic extraction reagent kit (Thermo Scientific) according to the manufacturer's instructions.

### Reporter gene assays.

For reporter gene assays, A549-NF-κB-LUC cells (see “Cells, viruses, and reagents” above) were seeded in 96-well plates 24 h prior to infection. Cells were lysed in passive lysis buffer (Promega), and the firefly luciferase activity was measured using a FLUOstar luminometer (BMG). The fold induction of NF-κB reporter activity was calculated by normalizing each result to the luciferase activity of the nonstimulated mock-infected control cells. For conventional reporter gene assays in HEK293T cells, cells were seeded in 96-well plates and transfected with 60 ng pNF-κB-LUC (R. Hofmeister, University of Regensburg, Regensburg, Germany) and 10 ng pTK-Ren (Promega) using polyethylenimine (PEI; Sigma-Aldrich), according to the manufacturer's protocol, 24 h prior to infection with the vv811 recombinant viruses. Cells were lysed, and firefly and renilla luciferase activities were measured as described above. In each case, the firefly luciferase activity was normalized to the renilla luciferase activity and the fold induction was calculated by normalizing each result to that for the nonstimulated mock-infected control cells. Experiments were performed in quadruplicate and repeated at least 3 times.

### Real-time PCR.

RNA was extracted from A549 cells grown in 12-well plates using an RNeasy RNA extraction kit (QIAgen) according to the manufacturer's protocol. Five hundred nanograms RNA was used to synthesize cDNA using SuperScript III reverse transcriptase (Invitrogen), also according to the manufacturer's protocol. cDNA was diluted 1:5 in water, and 2 μl was used as the template for real-time PCR using SYBR green PCR master mix (Applied Biosystems) and a Viia7 real-time PCR machine (Applied Biosystems). Expression of each gene was normalized to that of an internal control (GAPDH [glyceraldehyde-3-phosphate dehydrogenase]), and these values were then normalized to the value for the nonstimulated mock-infected control cells to yield the fold induction. The following primers were used: hGAPDH_fwd (5′ ACC CAG AAG ACT GTG GAT GG 3′), hGAPDH_rev (5′ TTC TAG ACG GCA GGT CAG GT 3′), hICAM-1_fwd (5′ TCT GTG TCC CCC TCA AAA GTC 3′), hICAM-1_rev (5′ GGG GTC TCT ATG CCC AAC AA 3′), hCCL2_fwd (5′ CAG CCA GAT GCA ATC AAT GCC 3′), hCCL2_rev (5′ TGG AAT CCT GAA CCC ACT TCT 3′), hCCL5_fwd (5′ CCC AGC AGT CGT CTT TGT CA 3′), hCCL5_rev (5′ TCC CGA ACC CAT TTC TTC TCT 3′), hIL-6_fwd (5′ AAA TTC GGT ACA TCC TCG ACG 3′), hIL-6_rev (5′ GGA AGG TTC AGG TTG TTT TCT 3′), hMxA_fwd (5′ ATC CTG GGA TTT TGG GGC TT 3′), hMxA_rev (5′ CCG CTT GTC GCT GGT GTC G 3′), VACVDNApol_fwd (5′ ATG GAT GTT CGG TGC ATT AA 3′), and VACVDNApol_rev (5′ GCA TTA AAT GGA GGA GGA GA 3′).

### ELISA.

Cell culture supernatants from vv811 recombinant virus-infected and TNF-α/IL-1β-stimulated A549 cells grown in 12-well plates were assayed for CCL5 and IL-6 protein using Duoset enzyme-linked immunosorbent assay (ELISA) reagents (R&D Biosystems) according to the manufacturer's instructions.

### Statistical analysis.

Data were analyzed using an unpaired Student's *t* test with Welch's correction where appropriate.

## RESULTS

### Construction of a recombinant vv811 lacking *A49R*.

**FIG 1:**
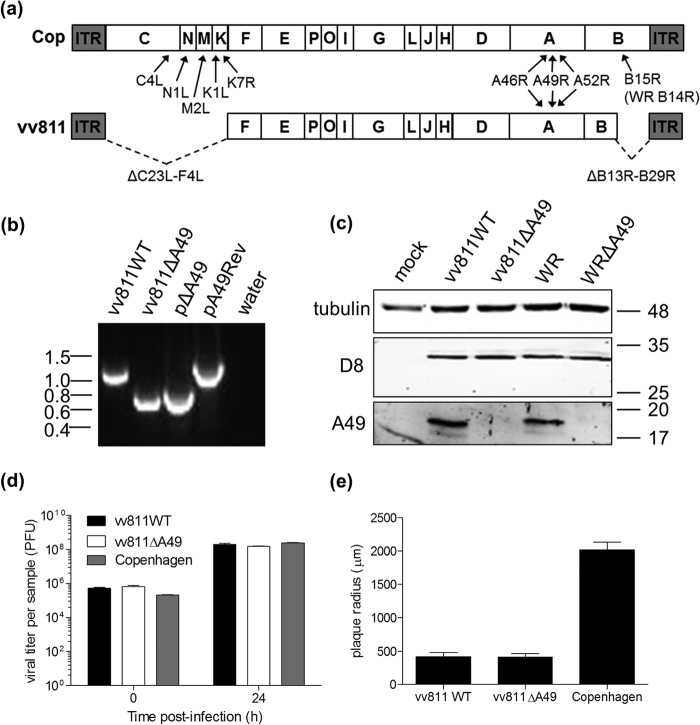
Construction of vv811 lacking *A49R*. (a) Schematic of the genome structure of VACV strains vv811 and Copenhagen (Cop), with the position of the known intracellular NF-κB inhibitors downstream of TNF-α or IL-1β indicated. ITR, inverted terminal repeat. (b) The *A49R* gene from vv811 was deleted by transient dominant selection (see Materials and Methods) to yield vv811ΔA49. A matching wild-type virus (vv811WT) was isolated from the same intermediate virus. The phenotype of the resolved viruses was analyzed by PCR from proteinase K-treated infected BSC-1 cell lysates using primers annealing to the flanking regions of *A49R*. The resulting PCR product sizes were compared to those obtained from the plasmid templates (pΔA49 and pA49Rev). Molecular mass markers (in kbp) are indicated on the left. (c) Expression of A49 by the recombinant vv811 viruses in lysates of BSC-1 cells mock infected or infected for 16 h with 2 PFU per cell was analyzed by immunoblotting using an anti-A49 polyclonal antiserum and anti-tubulin and anti-D8 antibodies as controls. Molecular mass markers (in kDa) are indicated on the right. (d) A549 cells were infected in duplicate with vv811 recombinant viruses or Copenhagen at 5 PFU per cell. Cells were then harvested at 0 h and 24 h, and the infectious titer of intracellular virus was determined by plaque assay on BSC-1 cells. (e) BSC-1 cells were infected with 50 PFU per well of vv811 recombinant viruses or Copenhagen, and plaques were allowed to form for 5 days. Cells were then stained with crystal violet, and the plaques were imaged and measured. Data are represented as the mean plaque radius (μm) ± SD.

The deletion strain of VACV, vv811, lacks 55 ORFs in the left and right terminal regions of the genome. Fagan-Garcia and Barry (2011) demonstrated that although vv811 lacked all known TNF-α-induced NF-κB inhibitors (Fig. 1a), this virus still inhibited NF-κB activation following stimulation of cells with TNF-α or IL-1β (30). Since then, two further VACV proteins have been characterized to inhibit NF-κB activation: C4 (21) and A49 (24). Although *C4L* is not present in the genome of vv811, *A49R* is (Fig. 1a), leading us to hypothesize that this protein was the cause of the NF-κB-inhibitory activity of vv811. To investigate this possibility, a mutant of vv811 lacking the *A49R* gene (vv811ΔA49), as well as a matching wild-type (WT) virus that was derived from the same intermediate virus (vv811WT), was generated by transient dominant selection (31) (see Materials and Methods). The genotype of the recombinant viruses was assessed by PCR analysis using primers that annealed to the flanking regions of the *A49R* gene (24). As expected, a product of approximately 600 bp (corresponding to the flanking regions only) was amplified from pΔA49- and vv811ΔA49-infected cells, indicating the loss of the *A49R* gene (Fig. 1b).

The lack of A49 expression by vv811ΔA49 was confirmed by immunoblotting analysis. Lysates of BSC-1 cells that had been infected with either the vv811 recombinant viruses or VACV strain Western Reserve (WR) expressing A49 or lacking A49 (WRΔA49) (24) were subjected to SDS-PAGE and immunoblotting analysis using an anti-A49 polyclonal antiserum (24). A49 expression was detected in cells infected with vv811WT and WR but not in cells infected with vv811ΔA49 or WRΔA49 (Fig. 1c). The levels of the late VACV protein D8 (35) expressed by vv811WT and vv811ΔA49 were equivalent, indicating equal infection by these viruses (Fig. 1c). Furthermore, the loss of A49 did not alter viral replication or spread in cell culture, because the viral titer in A549 cells after 24 h (Fig. 1d) and the size of the plaques formed at both 5 days (Fig. 1e) and 7 days (data not shown) postinfection by vv811WT and vv811ΔA49 were not significantly different. Although the replication of the vv811 viruses over a 24-h period in A549 cells was not significantly different from that of Copenhagen (Fig. 1d), the plaques in both BSC-1 cells (Fig. 1e) and A549 cells (data not shown) were significantly smaller than those formed by Copenhagen.

### pIκBα does not accumulate in cells infected with vv811ΔA49.

**FIG 2:**
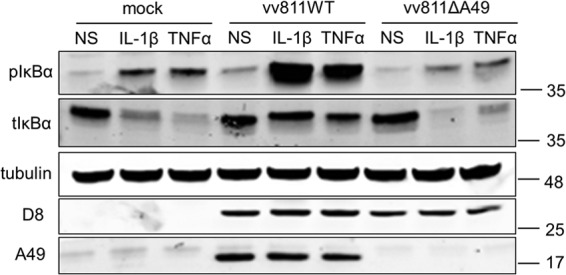
pIκBα does not accumulate in vv811ΔA49-infected cells. A549 cells were mock infected or infected for 16 h with 2 PFU per cell of the indicated viruses and then stimulated for 30 min with IL-1β (25 ng/ml) or TNF-α (100 ng/ml) or nonstimulated (NS) as a control by incubation with medium alone. The cells were lysed under conditions for detection of phosphorylated proteins. Lysates were analyzed by SDS-PAGE and immunoblotted using antibodies against the phosphorylated form of IκBα (pIκBα), total IκBα (tIκBα), tubulin, or D8 or with an anti-A49 polyclonal antiserum. Molecular mass markers (in kDa) are indicated on the right.

Recently, A49 was described to interact with β-TrCP and thus prevent the ubiquitination and subsequent degradation of pIκBα, causing pIκBα to accumulate (24). Previously, it was observed that pIκBα accumulated in cells infected with vv811 and stimulated with TNF-α/IL-1β (30). To address whether pIκBα still accumulated in cells infected with vv811 lacking A49, A549 cells were infected with the vv811 recombinant viruses and stimulated for 30 min with TNF-α/IL-1β to induce the activation of NF-κB, and pIκBα was measured by immunoblotting. In agreement with the findings of Fagan-Garcia and Barry (2011), there was a striking accumulation of pIκBα in response to both TNF-α and IL-1β in vv811WT-infected cells (Fig. 2). However, this accumulation was lost in cells infected with vv811ΔA49, where the levels of pIκBα were similar to those in mock-infected cells. These data indicate that in the absence of A49, vv811 no longer prevents the degradation of IκBα. Furthermore, this confirms the finding that although the sequence of A49 from WR (encoded by gene *175*), the strain of VACV with which the function of A49 was originally characterized, differed from the sequences of both Copenhagen and vv811 by a single amino acid at position 148 (a glutamic acid in WR and a lysine in Copenhagen and vv811), the function of the A49 protein was conserved among these strains.

### vv811ΔA49 is unable to inhibit p65 translocation in response to TNF-α and IL-1β stimulation.

**FIG 3:**
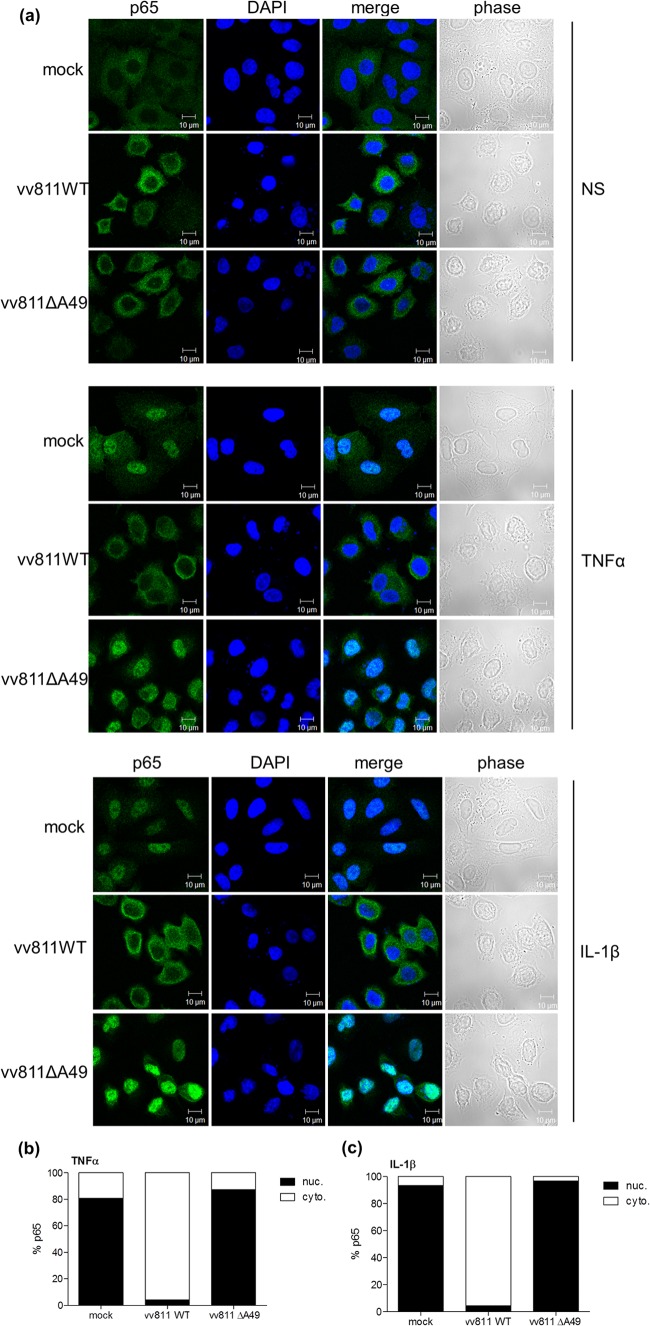
vv811ΔA49 does not inhibit p65 translocation in response to TNF-α or IL-1β stimulation. (a) A549 cells were mock infected or infected for 6 h with 5 PFU per cell of the indicated viruses and then stimulated for 30 min with TNF-α (50 ng/ml) or IL-1β (12.5 ng/ml) or nonstimulated (NS) as a control by incubation with medium alone. The cells were fixed and stained for immunofluorescence analysis using an antibody against intracellular p65 (green). Nuclei and viral factories were visualized by DAPI staining (blue). Merged and phase-contrast images are also shown. (b, c) The percentage of cells in which p65 was either cytoplasmic (cyto.) or nuclear (nuc.) was calculated from 250 to 300 cells for each condition in panel a.

**FIG 4:**
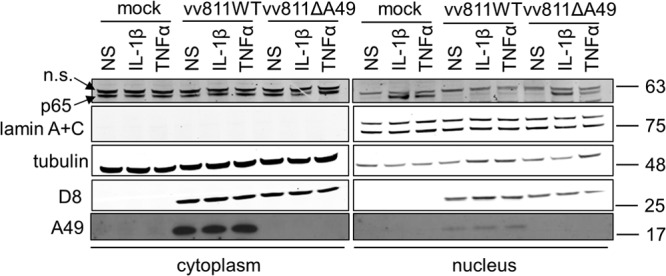
vv811ΔA49 does not inhibit p65 translocation in response to stimulation of cells with NF-κB agonists. A549 cells were mock infected or infected for 16 h with 2 PFU per cell of the indicated viruses and then stimulated for 30 min with IL-1β (25 ng/ml) or TNF-α (50 ng/ml) or nonstimulated (NS) as a control by incubation with medium alone. The cells were lysed and fractionated using a nuclear-cytoplasmic cell fractionating kit (Pierce). Cellular fractions (10% of the total nuclear fraction and 5% of the total cytoplasmic fraction) were analyzed by SDS-PAGE and immunoblotting using antibodies against the indicated proteins. Molecular mass markers (in kDa) are indicated on the right. n.s., nonspecific.

Given that IκBα was degraded in vv811ΔA49-infected cells that had been stimulated with either TNF-α or IL-1β, we next addressed whether p65 translocated into the nucleus in these cells. This question was investigated using two methods. First, A549 cells were infected for 6 h with the vv811 recombinant viruses and then stimulated for 30 min with either TNF-α or IL-1β to induce the translocation of p65 into the nucleus. Cells were then fixed, stained for endogenous p65, and analyzed by immunofluorescence microscopy. As expected, p65 translocated into the nucleus in mock-infected cells that had been stimulated with TNF-α or IL-1β, and this was inhibited in the presence of vv811WT infection, as described previously (30) (Fig. 3). However, this inhibition was lost in cells infected with vv811ΔA49, where p65 was present in the nucleus. Staining of cells with DAPI demonstrated the presence of viral factories in both vv811WT- and vv811ΔA49-infected cells. The amount of stimulant used was titrated down to find the lowest possible concentration sufficient to induce p65 translocation in nearly all cells. The same result was obtained when the length of infection was extended to 16 h (data not shown). Second, p65 translocation was assessed by biochemical fractionation of infected and TNF-α-/IL-1β-stimulated cells followed by immunoblotting analysis. In agreement with the results obtained by immunofluorescence, p65 was present in the nuclear fraction in response to both TNF-α and IL-1β stimulation in mock- or vv811ΔA49-infected cells but not in vv811WT-infected cells (Fig. 4). Immunoblotting of cellular fractions with antibodies against lamins A and C (found only in the nuclear fraction) and tubulin (found predominantly in the cytoplasmic fraction) confirmed the integrity of the cellular fractionation technique used. Taken together these results indicated that, unlike vv811WT, vv811ΔA49 lost the ability to inhibit p65 translocation downstream of TNF-α and IL-1β stimulation.

### vv811ΔA49 inhibits NF-κB-induced gene expression.

**FIG 5:**
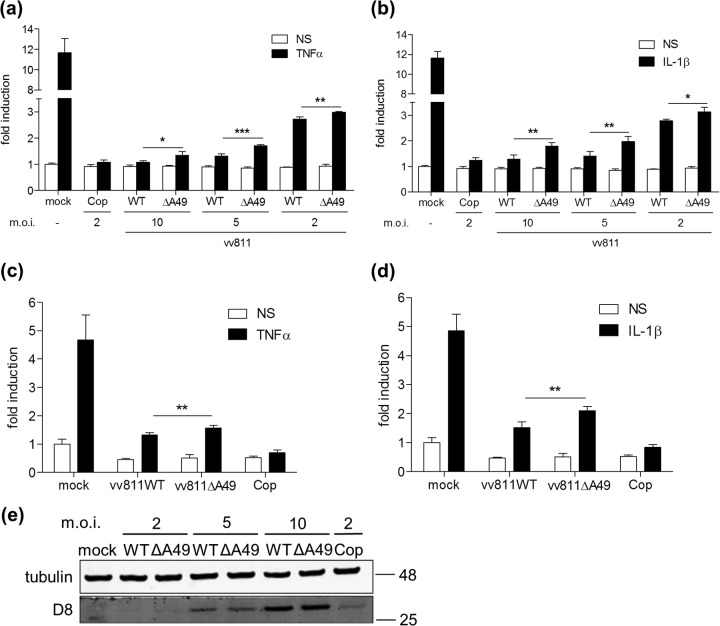
vv811ΔA49 inhibits TNF-α- and IL-1β-induced NF-κB-dependent reporter transcription. (a, b) A549-NF-κB-LUC cells were mock infected or infected in quadruplicate for 6 h at the indicated multiplicity of infection (m.o.i.) and then stimulated for 6 h with TNF-α (50 ng/ml) (a) or IL-1β (20 ng/ml) (b) or nonstimulated (NS) as a control by incubation with medium alone. (c, d) HEK293T cells were transfected in quadruplicate with 60 ng pNF-κB-LUC and 10 ng pTK-Ren using PEI. Twenty-four hours later the cells were mock infected or infected for 6 h with vv811 viruses (5 PFU per cell) or Copenhagen (2 PFU per cell) and then stimulated for 6 h with TNF-α (50 ng/ml) (c) or IL-1β (20 ng/ml) (d) or nonstimulated (NS) as a control by incubation with medium alone. The cells were lysed in passive lysis buffer, and the luminescence of each sample was measured and normalized to that of the nonstimulated mock-infected cells to give the fold induction. Data are shown as the mean ± SD and are representative of those from three experimental repeats. Significant differences between groups, determined using an unpaired Student's *t* test, are shown (*, *P* < 0.05; **, *P* < 0.01; ***, *P* < 0.001). (e) Protein expression from the reporter gene assay in A549 cells was analyzed by SDS-PAGE and immunoblotting using an antibody against the VACV D8 protein. Molecular mass markers (in kDa) are indicated on the right.

As p65 was able to translocate into the nucleus in vv811ΔA49-infected cells that had been stimulated with TNF-α/IL-1β, we next examined whether NF-κB-dependent gene transcription occurred in these cells. To do this we generated an A549-NF-κB-LUC cell line by transducing A549 cells with a lentivirus encoding the firefly luciferase gene under the control of an NF-κB-dependent promoter (see methods). These cells were then infected with either the vv811 recombinant viruses or VACV strain Copenhagen for 6 h and were subsequently stimulated with TNF-α/IL-1β for a further 6 h. As expected, due to the expression of a large number of NF-κB inhibitors, infection of A549-NF-κB-LUC cells with VACV Copenhagen inhibited NF-κB reporter activity in response to TNF-α (Fig. 5a) and IL-1β (Fig. 5b) stimulation. As vv811WT has the capacity to inhibit p65 translocation in response to these stimuli, infection with this virus also inhibited NF-κB reporter activity, and the level of inhibition correlated with the amount of virus that was used to infect the cells (Fig. 5a and b). Surprisingly, however, infection of cells with vv811ΔA49 also reduced NF-κB reporter activity, despite the inability of this virus to inhibit p65 translocation in response to stimulation. Although the level of NF-κB reporter inhibition in vv811ΔA49-infected cells was statistically significantly less than that in vv811WT-infected cells, due to the loss of A49, this difference was surprisingly small. The level of infection in these cells was confirmed by immunoblotting for D8 and was similar in vv811WT- and vv811ΔA49-infected cells (Fig. 5e). Similar NF-κB-inhibitory activity by vv811WT and vv811ΔA49 was also observed by conventional reporter gene assay in HEK293T cells transfected with an NF-κB-LUC reporter plasmid (Fig. 5c and d). To ensure that the observed NF-κB-inhibitory activity was not due to a cytotoxic effect induced by infection with the vv811 viruses, we assessed the viability of both A549 and HEK293T cells that had been infected for 12 h and found no reduction in viability compared to that of mock-infected cells (data not shown). Furthermore, the renilla luciferase activity in vv811WT- or vv811ΔA49-infected cells was not lower than that in mock-infected HEK293T cells after a 12-h infection (data not shown), indicating that the inhibition of NF-κB by these viruses is not due to a general shutdown of transcription or translation by the virus.

### vv811ΔA49 inhibits the expression of NF-κB-responsive cytokines and other gene transcripts.

**FIG 6:**
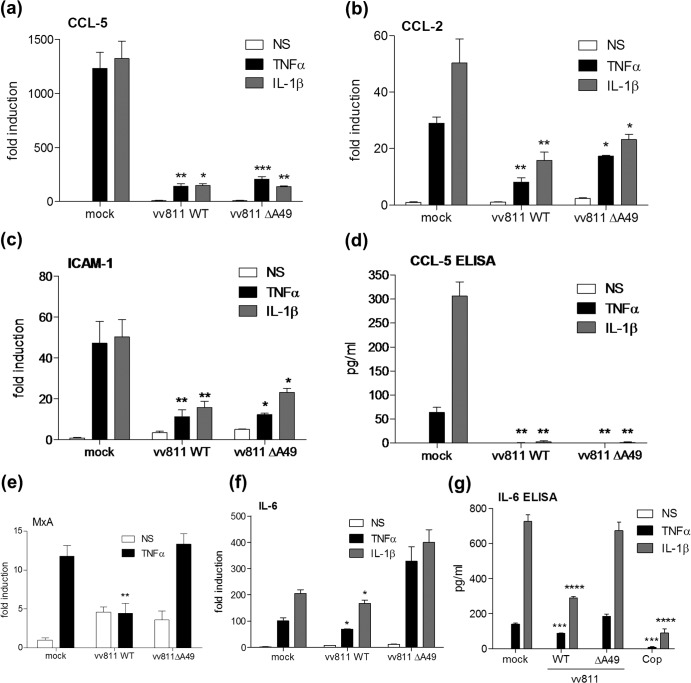
vv811ΔA49 inhibits TNF-α- and IL-1β-induced gene transcription and protein expression. A549 cells were mock infected or infected in triplicate for 6 h with 5 PFU per cell of the indicated viruses and then stimulated for 4 to 6 h with TNF-α (50 ng/ml) or IL-1β (25 ng/ml) or nonstimulated (NS) as a control by incubation with medium alone. The cell supernatant was carefully removed for ELISA, and the cells were lysed for RNA extraction. cDNA was then synthesized and used for quantitative PCR analysis. Expression of the genes for CCL-5 (a), CCL-2 (b), ICAM-1 (c), MxA (e), and IL-6 (f) was normalized to that of the gene for an internal control (GAPDH). These values were then normalized to those for the nonstimulated mock-infected cells, yielding the fold induction. The levels of the CCL-5 (d) and IL-6 (g) proteins in the cell supernatants were measured by ELISA. Data are shown as the mean ± SD and are representative of those from three experimental repeats. Significant differences between infected cells and the mock-infected control, determined using an unpaired Student's *t* test, are shown (*, *P* < 0.05; **, *P* < 0.01; ***, *P* < 0.001).

The inhibitory activity of the vv811 recombinant viruses was tested further by real-time PCR and ELISA analyses on infected and TNF-α/IL-1β-stimulated A549 cells. Consistent with reporter gene assay data, both vv811WT and vv811ΔA49 inhibited the transcription of the NF-κB-responsive genes for CCL-5 (Fig. 6a), CCL-2 (Fig. 6b), and intercellular adhesion molecule 1 (ICAM-1) (Fig. 6c) in response to both TNF-α and IL-1β. Furthermore, the level of CCL-5 protein production by these cells was inhibited by both viruses (Fig. 6d). Transcription of the viral DNA polymerase in these samples was also measured by real-time PCR and was found to be similar for the two viruses, indicating that the cells were infected with equivalent titers (data not shown). Upon further analysis of other NF-κB-responsive genes, it was noted that although vv811WT inhibited *MxA* (Fig. 6e) and *IL-6* (Fig. 6f) gene transcription, vv811ΔA49 failed to do so, suggesting that some genes are more susceptible to the NF-κB-inhibitory activity of this virus. These results for IL-6 were confirmed at the protein level by ELISA (Fig. 6g). Importantly, these data demonstrate that the inhibition of NF-κB-dependent gene expression and protein production by vv811ΔA49 is not due to a general translational shutoff by the virus, because production of IL-6 in vv811ΔA49-infected cells was equivalent to that in mock-infected cells. On the other hand, production of IL-6 was inhibited strongly by Copenhagen infection (Fig. 6g).

### Inhibition of NF-κB-induced gene expression by vv811ΔA49 requires only early gene expression.

**FIG 7:**
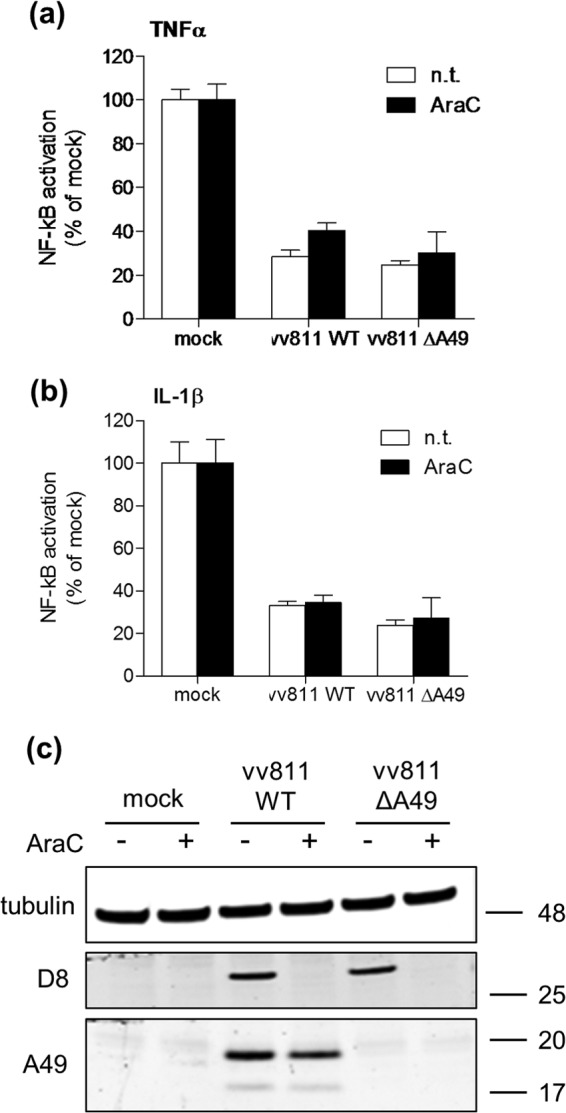
Inhibition of TNF-α- and IL-1β-induced NF-κB-dependent gene transcription by vv811ΔA49 does not require the expression of late viral proteins. (a, b) A549-NF-κB-LUC cells were mock infected or infected in quadruplicate for 6 h with 5 PFU per cell of virus either treated (AraC) or not treated (n.t.) with AraC at 40 μg/ml and then stimulated for 6 h with TNF-α (50 ng/ml) (a) or IL-1β (20 ng/ml) (b) or nonstimulated (NS) as a control by incubation with medium alone. The cells were lysed in passive lysis buffer, and the luminescence of each sample was measured and normalized to that for the nonstimulated mock-infected cells to give the fold induction. Data are shown as the mean ± SD and are representative of those from three experimental repeats. (c) Protein expression was analyzed by SDS-PAGE and immunoblotting using antibodies against the indicated proteins. Molecular mass markers (in kDa) are indicated on the right.

To investigate at which stage during the VACV replication cycle the NF-κB inhibitor was expressed, the reporter gene assay in A549-NF-κB-LUC cells was repeated in the presence of cytosine arabinoside (AraC), an inhibitor of viral DNA replication and late gene expression. As seen previously, both vv811WT and vv811ΔA49 inhibited NF-κB reporter activity downstream of TNF-α/IL-1β, and this inhibition still occurred under conditions where late viral gene expression was inhibited (Fig. 7a and b). Immunoblotting analysis confirmed the absence of the late VACV protein D8 (35) in the A549 cells infected in the presence of AraC (Fig. 7c). Together, these data indicate that the expression of an early viral gene or genes is responsible for the inhibition of TNF-α/IL-1β-induced NF-κB reporter activity by vv811ΔA49.

## DISCUSSION

The vv811 strain of VACV lacking 55 ORFs in regions of the viral genome rich in immunomodulators is a useful tool for the discovery of novel inhibitors of innate immunity and, in particular, those that target NF-κB. In addition to lacking the expression of the viral IL-1β receptor, encoded by *B15R* (36, 37), and the viral TNF receptor, encoded by the gene *A53R* (38), this virus also lacks the majority of other characterized inhibitors of NF-κB downstream of TNF-α/IL-1β (namely, K7, B14, N1, C4, K1, and M2; Fig. 1a). The only remaining inhibitors are A52 and A46. Fagan-Garcia and Barry (2011) suggested that these proteins are unlikely to be functional in vv811 and contain a number of mutations that may explain these observations (30). These authors also demonstrated that despite these multiple gene losses, vv811 prevented TNF-α/IL-1β-induced IκBα degradation and p65 translocation, suggesting the presence of a further inhibitor(s) (30). Given that the A49 protein was recently shown to inhibit NF-κB activation (24) and the *A49R* gene is present in vv811, we hypothesized that A49 might represent the NF-κB inhibitor of vv811, and a recombinant virus lacking *A49R* (vv811ΔA49) was generated to test this (Fig. 1).

Fagan-Garcia and Barry (2011) noted a large accumulation of pIκBα in TNF-α/IL-1β-stimulated vv811-infected cells and that some late viral protein expression contributed to this inhibition (30). In this study, we demonstrate that this accumulation of pIκBα is due to A49, because it was not observed in vv811ΔA49-infected cells (Fig. 2). A49 is expressed both early and late during infection, and its expression is reduced in the presence of AraC (24), thus explaining why the accumulation of pIκBα observed by Fagan-Garcia and Barry (2011) was less profound in the absence of late viral protein expression. Following degradation of pIκBα in vv811ΔA49-infected cells, p65 translocated into the nucleus (Fig. 3 and 4), indicating that this virus expresses no additional proteins that can block NF-κB activation up to and including p65 translocation. Despite the presence of p65 in the nucleus, vv811ΔA49 retained the ability to inhibit NF-κB reporter activity almost as well as vv811 (Fig. 5) and did so in the presence of AraC (Fig. 7). This inhibition was independent of the well-known inhibition of host protein synthesis caused by VACV infection (39, 40), because some proteins, like IL-6 and renilla luciferase, were still synthesized (Fig. 6). These findings reveal the presence of yet another VACV inhibitor targeting NF-κB activity which is expressed early during infection and acts downstream of p65 translocation.

Recently, VACV protein N2 was described to be a novel virulence factor that inhibits the transcription factor IRF-3 in the nucleus (34). Therefore, it is tempting to suggest that this remaining NF-κB inhibitor(s) exerts its effect in the nucleus. Examples of such an inhibitor are present in other viruses. Protein A238L of African swine fever virus, for example, prevents the activation of NF-κB, as well as that of NF-ATc2 and c-Jun, through an interaction with the transcriptional coactivator p300 (41). Furthermore, the retroviral cyclin protein of walleye dermal sarcoma virus inhibits NF-κB-dependent transcription via an interaction with TATA-binding protein-associated factor 9 (TAF9) (42). Interestingly, myxoma virus expresses a protein encoded by the *M150R* gene that localizes with NF-κB in the nucleus and inhibits NF-κB activation by an as yet unknown mechanism (43). An ortholog of this protein (encoded by *C9L*) exists in VACV, but it is not present in the genome of vv811 and an anti-NF-κB function has not yet been ascribed to it. Finally, gene *002* of the parapoxvirus orf virus encodes a protein that reduces the acetylation of p65 and results in a reduced interaction of p65 with p300 (44). An ortholog of this protein has not been identified in VACV. Despite these examples, it remains possible that the unidentified VACV inhibitor modifies p65 in some manner in the cytoplasm that still allows it to translocate but negatively impacts transcription. The identity of this remaining inhibitor and the exact mechanism by which it targets NF-κB-dependent transcription require further investigation. However, given that vv811ΔA49 inhibits the activity of an NF-κB-dependent reporter (Fig. 5) and the transcription of certain NF-κB-responsive genes, such as the genes for CCL-5, CCL-2, and ICAM-1 but not those for IL-6 or MxA (Fig. 6), it is possible that the inhibitory action might be gene/promoter specific and may depend on the proteins recruited to these individual transcriptional complexes. Defining which gene subsets are inhibited by vv811ΔA49 will require a broader transcriptional analysis. Further, if this remaining unidentified inhibitor(s) is affecting the composition or activity of transcriptional complexes, it is possible that its activity may not be limited to NF-κB inhibition and perhaps other transcription factors are targeted as well.

The reason why VACV requires so many inhibitors of NF-κB activation is currently unclear but likely reflects the complex inflammatory environment and signaling that are induced following infection *in vivo*. This involves significant cross talk between different innate immune-signaling pathways (45) and cell type- and host-determined specificities, all of which may play a crucial role in the outcome of infection. This is supported by the lack of redundancy of VACV NF-κB inhibitors *in vivo*, as illustrated by the observation that in every case tested a single gene deletion caused attenuation of the virus (11, 14, 21, 24, 46–48). *In vitro* studies using strains of VACV with a large complement of innate immunomodulators, such as WR or Copenhagen, often fail to identify novel NF-κB inhibitors during infection due to the functional redundancy observed in cell culture. Deficient viruses, such as vv811, can therefore be attractive tools for the study of other aspects of host antiviral signaling, such as inhibition of IRF-3/7 activation. The identification of novel inhibitors of innate immunity is an important aspect of VACV research and, in particular, for vaccine design. This is highlighted by recent publications reporting the enhanced immunogenicity of VACV strains lacking the IL-1-binding protein (encoded by *B15R* [49]), IL-18-binding protein (encoded by *C12L* [50]), and type I IFN-binding protein (encoded by *B18R* [51]) and the intracellular IRF-3/7 inhibitor *C6L* (52, 53), all of which are absent in vv811, thus highlighting this virus as an attractive candidate for a novel vaccine vector.
